# Dengue in Araraquara, state of São Paulo: epidemiology, climate and *Aedes aegypti* infestation

**DOI:** 10.11606/S1518-8787.2018052000414

**Published:** 2018-02-07

**Authors:** Aline Chimello Ferreira, Francisco Chiaravalloti, Adriano Mondini

**Affiliations:** I Universidade Estadual Paulista “Júlio de Mesquita Filho”. Faculdade de Ciências Farmacêuticas. Programa de Pós-Graduação em Biociências e Biotecnologia Aplicadas à Farmácia. Araraquara, SP, Brasil; II Universidade de São Paulo. Faculdade de Saúde Pública. Departamento de Epidemiologia. São Paulo, SP, Brasil; III Universidade Estadual Paulista “Júlio de Mesquita Filho”. Faculdade de Ciências Farmacêuticas. Departamento de Ciências Biológicas. Araraquara, SP, Brasil

**Keywords:** Dengue, epidemiology, Disease Outbreaks, Risk Factors, Communicable Diseases, Epidemiological Surveillance, Dengue, epidemiologia, Surtos de Doenças, Fatores de Risco, Doenças Transmissíveis, Vigilância Epidemiológica

## Abstract

**OBJECTIVE:**

To describe the epidemiology of dengue in a medium-sized city in the state of São Paulo.

**METHODS:**

Data, such as circulating serotypes, severe cases and deaths, age group, sex, among others, were obtained on reported and confirmed dengue cases in Araraquara, state of São Paulo, between 1991 and 2015. Climatic and infestation data were also analyzed. These variables were evaluated descriptively, using statistical measures such as frequencies, averages, minimum and maximum. Dengue incidence rates were calculated according to month, year, age and sex, and time series of dengue cases, infestation, and climatic variables.

**RESULTS:**

Approximately 16,500 cases of dengue fever were reported between 1991 and 2015. The highest number of reports was recorded in 2015 (7,811 cases). In general, the age group with the highest number of reports is between 20 and 59 years old. The highest incidences, generally between March and May, occurred after the increase in rainfall and infestation in January.

**CONCLUSIONS:**

Increased levels of infestation due to rainfall are reflected in incidence rates of the disease. It is fundamental to know the epidemiology of dengue in medium-sized cities. Such information can be extended to diseases such as Zika and Chikungunya, which are transmitted by the same vector and were reported in the city. The intensification of surveillance efforts in periods before epidemics could be a strategy to be considered to control the viral spread.

## RESUMO

**OBJETIVO:**

Descrever a epidemiologia da dengue em cidade de médio porte do estado de São Paulo.

**MÉTODOS:**

Foram obtidas informações sobre os casos notificados e confirmados de dengue em Araraquara, SP, entre 1991 e 2015, como sorotipos circulantes, casos graves e óbitos, faixa etária, sexo, entre outras. Também foram levantadas as informações climáticas e de infestação pelo vetor. Essas variáveis foram trabalhadas descritivamente, utilizando-se medidas estatísticas como frequências, médias, mínimo e máximo. Calcularam-se taxas de incidência de dengue segundo mês, ano, idade e sexo, e série histórica de casos de dengue, infestação e variáveis climáticas.

**RESULTADOS:**

Foram confirmados 16.431 casos de dengue entre 1991 e 2015. O maior número de notificações foi registrado em 2015 (7.811 casos). De forma geral, a faixa etária com o maior número de notificações encontra-se entre 20 e 59 anos. As maiores incidências, geralmente entre março e maio, ocorreram após o aumento da pluviosidade e infestação, em janeiro.

**CONCLUSÕES:**

Os altos níveis de infestação que aumentam com as chuvas refletem-se nas taxas de incidência da doença. É fundamental conhecer a epidemiologia da dengue em cidades de médio porte. Seus resultados podem ser estendidos para doenças como Zika e Chikungunya (transmitidas pelo mesmo vetor e notificadas na cidade). A intensificação dos esforços de vigilâncias em períodos que precedem as epidemias poderia ser uma estratégia a ser considerada para o controle da dispersão viral.

## INTRODUCTION

Dengue is a viral disease that is a major public health problem. It entails 20 thousand deaths and 500 thousand hospitalizations annually. In addition, 390 million people are infected each year, but only 96 million cases are symptomatic^5^ ^,^ ^20^. The disease is caused by one of the four serotypes of the dengue virus (DENV 1-4), which are transmitted by mosquitoes of the genus *Aedes* ^39^. *Aedes aegypti* is the most important vector of dengue, zika, and chikungunya in Brazil^34^. The genus *Flavivirus, Flaviviridae* family, circulates in Asia, Africa, the Americas and more recently in Europe^13^.

The spatial distribution of these vectors strongly affects the epidemiology of the disease. In addition, the life cycle of *Ae. aegypti* is almost completely dependent on the environments created by humans and varies according to changes and climatic fluctuations^14^. The increase in temperature, variations in rainfall and relative air humidity favor the number of breeding sites available and the development of the vector^35^.

Dengue is a global concern, and the current trends are: rapidly expanding the geographical distribution of the vector and spread of the virus. In addition, the continuous circulation of the four serotypes is associated with the magnitude of the epidemics and the increase of severe manifestations and deaths due to infection. Reports of dengue fever in Brazil have increased dramatically since the 1980s. Rapid and unplanned urbanization poor living conditions, and inefficiency of surveillance and vector control are some of the factors related to the dispersion of DENV^3^ ^,^ ^11^.

In addition to dengue, chikungunya (CHIKV) and zika (ZIKV) are equally worrying arboviruses in expansion in Brazil. The first belongs to the *Togaviridae* family, and was reported in the country in September 2014. It affected 2,772 people in the North and Midwest regions that same year. ZIKV is also a *Flavivirus* that dispersed throughout the country and were first reported in Bahia, in April 2015^34^.

The increase in the number and severity of dengue cases in Brazil and worldwide demands investigations to identify patterns of transmission in cities with similar attributes. Knowing the epidemiological and clinical aspects of the disease in endemic areas is essential for the implementation of interventions to modify transmission patterns. The study of dengue fever and its relationship with vectors and climatic variables can also identify areas at risk for ZIKV and CHIKV infections. *Ae. aegypti* is known to be also a vector of these viruses, which makes it possible to map out common surveillance and control strategies^1^ ^,^ ^10^.

The objective of this study was to describe the epidemiology of dengue in a medium-sized city in the state of São Paulo.

## METHODS

Located in the southeastern region of Brazil, the state of São Paulo concentrates 22% of the Brazilian population. It is the most densely populated state in the country and the third most populous political unit in South America^12^. São Paulo is divided into 645 municipalities and has the second highest Human Development Index (HDI) in Brazil.

Araraquara (21º47’40” south latitude, 48º10’32” west longitude) stands out, regionally and nationally, in terms of quality of life (HDI = 0.815). The city has an estimated population of 226,508 inhabitants, a territorial area of 109.88 km^2^ and 206.68 inhabitants/km^2^.^19^

**Figure 1:**
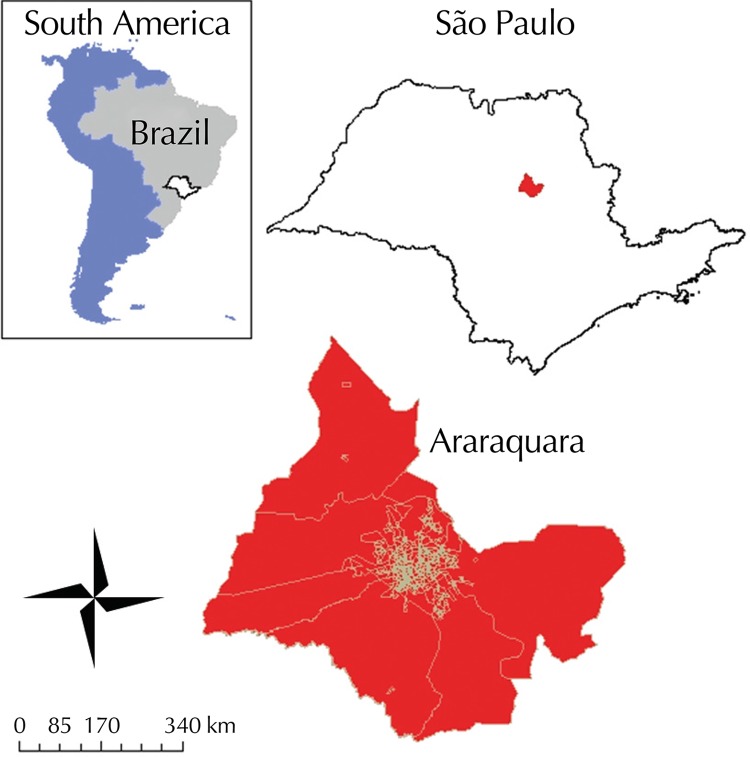
Location of the state of São Paulo in Brazil and South America. Location of the city of Araraquara in the state and its 422 census tracts in detail.

It is located in the center of São Paulo, 270 km from the capital. It is one of the most industrialized cities in the state. Its economy is focused on manufacturing (food, aeronautics, chemical-pharmaceutical and civil construction), agribusiness (sugar, ethanol and orange juice) and commerce and services, as well as on education (several public and private universities) (Figure 1).

A descriptive and retrospective epidemiological study was carried out to establish a profile of the dengue occurrence in Araraquara. Health-related aspects of human populations, such as age, sex, socioeconomic level, geographical location, among other variables^29^ were considered.

Dengue reports were recovered from (i) Epidemiological Surveillance Center website (CVE)^8^, for the cases occurred between 1991 and 2007; and (ii) with the Special Health Service of Araraquara (SESA), through the Information System on Diseases of Compulsory Declaration (SINAN)^24^, for cases occurring between 2008 and 2015. Demographic and socioeconomic information provided by the Brazilian Institute of Geography and Statistics (IBGE), as well as the geographical databases of Araraquara, São Paulo, Brazil and South America (Universal Transverse Mercator [UTM], zone 22S, WGS 1984)^18^.

The climatic information (average rainfall, humidity, and temperature) was collected monthly and yearly on the website of Centro Integrado de Informações Agrometeorológicas (CIIAGRO)^a^, Companhia Ambiental do Estado de São Paulo (CETESB)^b^ and Departamento Autônomo de Água e Esgotos de Araraquara (DAAE)^c^.

The positivity of the strategic points (StP) obtained from the Superintendência de Controle de Endemias (Sucen SR-06) was used as a measure of larval infestation. The StP are real estate with greater importance in the generation and active and passive dispersion of *Ae. aegypti*. Deposits of tires junkyard, automobile dismantling workshops, rubber shops, factories, cemeteries, transporters, road and rail stations, ports and airports are considered important strategic points. The positivity of these properties is calculated by the number of positive properties for *Ae. aegypti* (×100) divided by the number of properties that were assessed. These measures are carried out monthly^d^.

Time series were constructed from the data referring to the occurrence of dengue in Araraquara between 1991 and 2015. In the first series, the first imported case up to the autochthonous cases of 2015 were included in the analysis, as well as the circulating serotypes, severe cases, and deaths due to infection. We considered all dengue cases confirmed by laboratory or clinical-epidemiological criteria, as recommended by the Ministry of Health^24^.

Information from 1991 to 2007 was presented to provide a historical parameter of dengue transmission in the city. However, the secondary data were limited and could not be used in the descriptive analysis for this period.

Dengue cases reported between 2008 and 2015, climatic variables, and positivity of monthly StP were associated to generate a second time series. Cases of dengue were presented as percentages, dividing the number of cases of each month by the total number of cases of the respective year (×100). This procedure was used to standardize curves of cases per dengue-year, since there could be distortions if absolute numbers were used. The relationship between the occurrence of dengue, the entomological indexes, and the climatic variables could be visualized comparing the formats of the monthly curves.

Cases were organized by dengue-year, a criterion commonly used in epidemiological studies of dengue^25^. In this approach, data are analyzed considering cases reported between September of a year to August of the following year to evidence the seasonality of the disease.

Data were organized based on the date of the onset of the disease and analyzed according to gender, age, classification and evolution of the case (cure, death due to dengue, death due to other causes). We used World Health Organization revised classification of dengue which was adopted in 2014: dengue without warning signs, dengue with signs of alarm and severe dengue)^39^. Gross incidence rates per month and year were calculated for the entire period.

The incidence adjusted by age and sex was estimated for each year, applying the direct method^21^. This is the most common strategy to remove the effect of bias due to different age structures in different populations or in the same population at different periods. This adjustment was performed by multiplying incidence rates by age group with specific weights for the same range for each year. The weights within each age group were calculated with the proportion of São Paulo state population, based on 2010 demographic census and estimates of population per year. The adjustment of cases by age provided age-adjusted incidence rates of dengue for each year of the period.

## RESULTS

**Figure 2:**
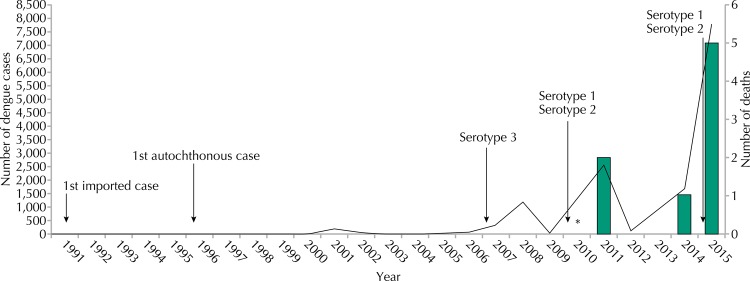
Time series of the dengue occurrence, the first reported case until 2015 and the number of deaths due to dengue. Araraquara, state of São Paulo, 1991 to 2015.

The first confirmed dengue case in Araraquara was imported and occurred in 1991 (Figure 2). Between 1991 and 2015, 16,431 cases were reported, and the first outbreak occurred in 2008. Severe cases and deaths were reported as of 2010. Information on circulating serotype(s) was not available until 2007. The detection of DENV-3 serotype in 2008, DENV-1 in 2010 and DENV-1, 2 and 3 in 2015, was performed through viral isolation of a small percentage of patients.

* First case of dengue with warning signs.

**Figure 3:**
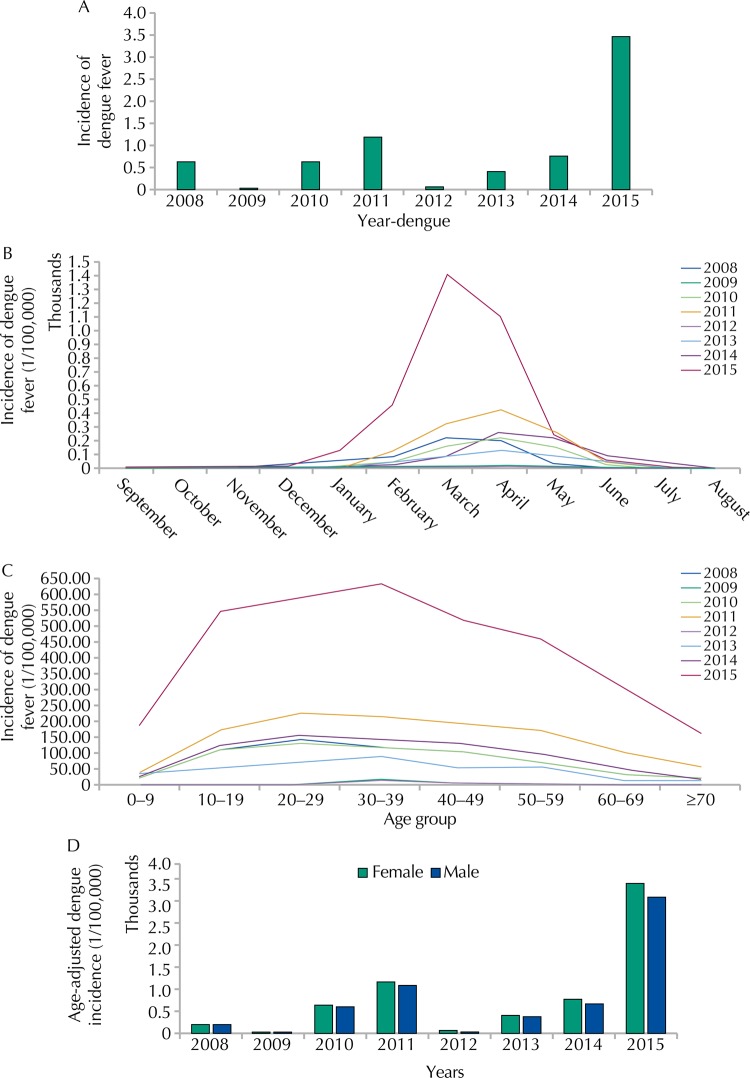
Annual and monthly distribution of dengue cases. Araraquara, state of São Paulo, Brazil, 2008–2015.

We recovered 15,729 dengue cases, distributed per dengue-year, between September 2007 and August 2015. The first dengue outbreak in 2008 had an incidence of 639 cases per 100,000 inhabitants. The most severe outbreak in the time series had an incidence of 3,448 cases per 100,000 inhabitants and occurred in 2015. The periods between March and May presented the highest incidences. However, an increase in dengue reports was noticed as of January. Autochthonous cases in all months of the year started to occur in 2008 (Figure 3, A and B).

A: incidence of dengue cases per year; B: incidence of dengue cases per month and year; C: annual incidence of dengue by age group; D: annual incidence of dengue by sex adjusted sex adjusted for age (incidences calculated per 100,000 inhabitants)

The most affected age group in the eight-year period was from 20 to 39 years (Figure 3, C). The age-adjusted incidence was slightly higher for females (Figure 3, D). However, a higher difference in this percentage was noticed for 2015.

**Figure 4:**
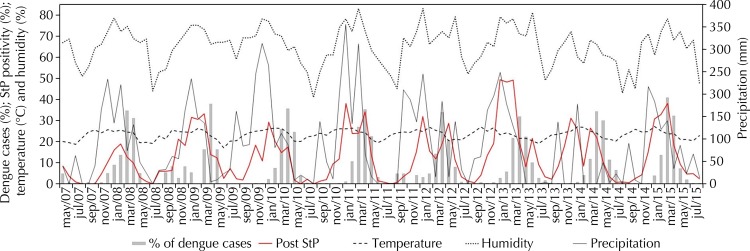
Time series of dengue occurrence per month, according to proportional distribution of dengue cases (%), positivity for strategic points (StP), mean temperature (ºC), mean relative humidity (%) and mean precipitation (mm). Araraquara, state of São Paulo, May 2007 to August 2015.

The increase of precipitation and humidity curves were followed by the positivity of strategic points (Figure 4). Precipitation and humidity growth were key factors for the increase of *Ae. aegypti* infestation. In addition, case curves increased one or two months after the peaks of rainfall and infestation. There is an apparent relationship between the increase of temperature and infestation growth and dengue cases, although this relation seems discrete.

## DISCUSSION

There was an increase in the number of months with dengue cases over the years, especially the ones considered interepidemic. Thus, we could describe the process of dengue endemization in the city. This process refers to the occurrence of dengue cases in every month of the year, with no introduction of serotypes. According to Mondini et al.^25^, endemization allows a continuous viral transmission, since the occurrence of dengue in the previous summer guarantees the occurrence of transmission in the following season.

The first major dengue outbreak occurred in São Paulo in 1990, resulting in viral circulation in a large portion of the state^2^. Although Araraquara was infested by *Ae. aegypti* since 1986, the first dengue epidemic in the city (2008) occurred late when compared to proximate cities. Ribeirão Preto^15^ and São José do Rio Preto, located respectively 93 km and 170 km away from Araraquara, experienced the first outbreaks in 1990^e^.

The growth of dengue cases in Araraquara in 2008 may be related to the introduction of DENV-3 in 2007. Differently from what occurred in Brazil and the state of São Paulo, where DENV-3 circulated since 2001^26^ ^,^ ^37^, this late introduction might have favored this outbreak, due to the susceptibility of the population to this serotype. The first severe cases occurred in 2010, when DENV-1 and 2 were circulating. The circulation of both serotypes influenced the next outbreak (2011).

The major epidemics in Araraquara were followed by outbreaks in neighboring municipalities such as Matão, Rincão, Motuca and Américo Brasiliense^8^. This trend reinforces its role as a disseminator or receptor of DENV. Araraquara is the most populated city in the micro-region^19^, and stands out economically for its commerce and education resources, causing intense human migration to the city. Roseghini et al.^32^ showed that socioeconomic factors favor the flow of people from smaller to larger cities in search of better health services, or for work and leisure. This migration of the population could play a key role in the occurrence of epidemics. This association is little known and deserves further studies.

Araraquara presented an expressive increase in number and severity of cases and deaths. Paixão et al.^27^ demonstrated the risk of death due to dengue increased significantly between 2000 and 2011 in all regions of Brazil. A study in Amazonas indicated that 88% of dengue-confirmed deaths had been identified by health services as severe cases. A more careful attention to signs of dengue could have contributed to reducing disease mortality^28^. A recent review indicates that early diagnosis and immediate treatment of severe cases may reduce disease severity and mortality^38^.

We did not investigate severe cases and deaths in particular, but the most affected age group in the period was between 20 and 59 years. This group corresponds to the economically active population, who works or studies during the day. Women presented a small difference in the distribution of cases and were more affected than men. The same patterns for age group and gender was observed in a study carried out in São José do Rio Preto^33^. A higher incidence in women from different regions may be related to the fact that they tend to seek more medical care than men. This observation may represent a bias in comparisons of incidence rates^7^.

Incidence rates in Araraquara presented seasonal trends, increasing after the growth of infestation, which was followed by the elevation of rainfall. The increment in case numbers from March to May have resulted from the rainfall in January and March. This indicates a pattern in which rainfall in one month causes an increase in the number of cases in the subsequent two months. A higher rainfall influenced the availability of oviposition sites^35^ and caused an increase in infestation that was observed. Several studies found a similar pattern between rainfall and dengue incidences^9^ ^,^ ^22^ ^,^ ^30^. These results may be useful for the development of policies for dengue control and prevention.

The association between temperature and infestation was mild and did not seem to influence incidence rates. In general, summer and winter presented high temperatures, which are adequate for vector proliferation. A study in the city of São Paulo demonstrated that dengue incidence was more affected by temperature^2^. The increase in temperature influenced the dynamics of *Ae. aegypti* and, consequently, the transmission of the dengue virus^4^ ^,^ ^31^.

According to Viana and Ignotti^35^, meteorological variables such as temperature, humidity and rainfall influence the vector dynamics and incidences of dengue in Brazil. Rainfall and temperature favor an increase in breeding sites and dengue cases. In dry and low-temperature periods, there is a decrease in the number of vectors, but it is not enough to stop the transmission of the disease, due to the avid hematophagous behavior of the vector which favors virus dispersion. Horta et al.^17^ have shown that models based on climatic variables that consider the interval between rainfall, temperature and dengue can be useful in dengue control programs in tropical countries.

Epidemiological surveillance of dengue is mainly based in monitoring the density of vector infestation, to avoid levels of infestation capable of sustaining transmission and monitoring human cases to detect the early onset of outbreaks^34^. In our study, we were able to demonstrate aspects that favor the occurrence of dengue in Araraquara. These aspects could be useful for the development of effective surveillance strategies resulting in adoption of more adequate control measures to reduce or slow the transmission. For effective surveillance, early recognition of local transmission is necessary, followed by rapid and effective vector control and other measures that incorporate the ecological, entomological and virological components^16^.

In Brazil, the major epidemics caused by these viruses occur due to the widespread infestation of vectors throughout the territory and the susceptibility of the population, which contributes to DENV dissemination^16^. Another key factor is the delay of the surveillance system in early recognizing transmission and initiating effective control measures in a timely manner. According to Viennet et al.^36^, the delay in reporting cases is a crucial determinant of DENV transmission. They suggest that future research should include analyses of areas with high rates people migration alongside demographic, socioeconomic and entomological factors. As seen in Araraquara, knowledge of DENV epidemiology provided information on the most appropriate moment to start dengue control programs. In addition, these actions could also be guidelines for surveillance and control of zika and chikungunya. Both viruses are transmitted by the same vector and presented autochthonous transmission in the city^8^.

This is the first study on dengue epidemiology in Araraquara, and like most descriptive epidemiological studies, presented limitations. These include the lack of information on cases reported prior to 2007; the fact that it was conducted using secondary data, and lack of molecular surveillance with differential diagnosis of circulating serotypes in the city. Despite these limitations, our study showed the importance of knowing the epidemiological aspects of dengue in medium-sized endemic municipalities, between 150 and 250 thousand inhabitants. Actions in these areas can guide actions in other municipalities with similar characteristics.

Kularatne^23^ concluded that the key to all prevention programs is surveillance to detect early outbreaks. This study showed that the incidence of dengue begins to increase in the month of January, which coincides with rain peaks. This increase in rainfall is reflected in the incidence rates in the subsequent months. This period may be indicative of when to start disease control measures. It is recommended that surveillance services initiate an active search for cases and areas infested by *Ae. aegypti* when the rainfall is identified. From there, vector control measures must be intensified in these areas, as well as the dissemination of these measures to the population through media campaigns and other means of communication. It is important to increase attention to the first symptoms of the disease and measures to eliminate water accumulation, avoiding the birth and proliferation of the mosquito. In addition, alerting the medical class to the symptoms of dengue, chikungunya, and zika in the interepidemic months would aid in the early detection of transmission. These actions could provide faster responses to contain transmission of these diseases and would innovate surveillance currently employed. Emergency control measures would be replaced by preventive surveillance, in which decreased infestation would result in lower incidences in epidemic periods.

## Footnotes

a Centro Integrado de Informações Aagrometeorológicas. São Paulo: CIIAGRO; s.d. [cited 2016 Sep 15]. Available from: http://www.ciiagro.sp.gov.br

b Companhia Ambiental de Estado de São Paulo. Sistema de Informações da Qualidade do Ar - QUALAR. São Paulo: CETESB; s.d. [cited 2015 Sep 15]. Available from: http://ar.cetesb.sp.gov.br/qualar/

c São Paulo (Estado), Secretaria de Saneamento e Recursos Hídricos, Departamento de Águas e Energia Elétrica. Hidrologia: banco de dados hidrológicos. São Paulo: DAEE; s.d. [cited 2016 Sep 15]. Available from: http://www.hidrologia.daee.sp.gov.br

d Secretaria de Estado de Saúde de São Paulo, Superintendência de Controle de Endemias. São Paulo: SUCEN; 2016 [cited 2017 Oct 26]. Available from: http://www.saude.sp.gov.br/resources/sucen/homepage/downloads/arquivos-dengue/norte.pdf

e Chiaravalloti-Neto F. Epidemiologia da dengue nas regiões de São José do Rio Preto e Araçatuba, São Paulo, 1990 a 1996 [these]. São Paulo: Faculdade de Saúde Pública da Universidade de São Paulo; 1999.
